# Gepotidacin for the Treatment of Uncomplicated Urogenital Gonorrhea: A Phase 2, Randomized, Dose-Ranging, Single-Oral Dose Evaluation

**DOI:** 10.1093/cid/ciy145

**Published:** 2018-04-02

**Authors:** Stephanie N Taylor, David H Morris, Ann K Avery, Kimberly A Workowski, Byron E Batteiger, Courtney A Tiffany, Caroline R Perry, Aparna Raychaudhuri, Nicole E Scangarella-Oman, Mohammad Hossain, Etienne F Dumont

**Affiliations:** 1 Section of Infectious Disease, Louisiana State University Health Sciences Center, New Orleans; 2 Desert AIDS Project, Palm Springs, California; 3 Department of Medicine, Division of Infectious Diseases, MetroHealth Medical Center, Cleveland, Ohio; 4 Department of Medicine, Division of Infectious Diseases, Emory University Department of Medicine, Atlanta, Georgia; 5 Department of Medicine, Division of Infectious Diseases, Indiana University School of Medicine, Indianapolis; 6 Research & Development, GlaxoSmithKline, Collegeville, Pennsylvania

**Keywords:** gepotidacin, urogenital, gonorrhea, *Neisseria gonorrhoeae*

## Abstract

**Background:**

In this phase 2 study, we evaluated the efficacy and safety of oral gepotidacin, a novel triazaacenaphthylene bacterial type II topoisomerase inhibitor, for the treatment of uncomplicated urogenital gonorrhea.

**Methods:**

Adult participants with suspected urogenital gonorrhea were enrolled and completed baseline (day 1) and test-of-cure (days 4–8) visits. Pretreatment and posttreatment urogenital swabs were collected for *Neisseria gonorrhoeae* (NG) culture and susceptibility testing. Pharyngeal and rectal swab specimens were collected if there were known exposures. Participants were stratified by gender and randomized 1:1 to receive a 1500-mg or 3000-mg single oral dose of gepotidacin.

**Results:**

The microbiologically evaluable population consisted of 69 participants, with NG isolated from 69 (100%) urogenital, 2 (3%) pharyngeal, and 3 (4%) rectal specimens. Microbiological eradication of NG was achieved by 97%, 95%, and 96% of participants (lower 1-sided exact 95% confidence interval bound, 85.1%, 84.7%, and 89.1%, respectively) for the 1500-mg, 3000-mg, and combined dose groups, respectively. Microbiological cure was achieved in 66/69 (96%) urogenital infections. All 3 failures were NG isolates that demonstrated the highest observed gepotidacin minimum inhibitory concentration of 1 µg/mL and a common gene mutation. At the pharyngeal and rectal sites, 1/2 and 3/3 NG isolates, respectively, demonstrated microbiological cure. There were no treatment-limiting adverse events for either dose.

**Conclusions:**

This study demonstrated that single, oral doses of gepotidacin were ≥95% effective for bacterial eradication of NG in adult participants with uncomplicated urogenital gonorrhea.

**Clinical Trials Registration:**

NCT02294682.

## Teaser

In this phase 2 study, single oral doses of gepotidacin were ≥95% effective for bacterial eradication in culture-proven uncomplicated urogenital gonorrhea. New antibiotics for drug-resistant *Neisseria gonorrhoeae* are urgently needed. With additional evaluation, gepotidacin may provide an alternative therapeutic option.

Gonorrhea is a sexually transmitted infection caused by *Neisseria gonorrhoeae* (NG), with 78 million infections reported globally in 2012 [1]. In the United States, approximately 468500 gonococcal infections were reported in 2016, an 18.5% increase from 2015 [2], while in Europe, approximately 66000 gonococcal infections were reported in 2014, a 25% increase from 2013 [3]. Consequences of untreated gonococcal infections include pelvic inflammatory disease, infertility in women and men, ectopic pregnancy, tubo-ovarian abscess, neonatal conjunctivitis, and disseminated gonorrhea [4].

Over the past few decades, NG has demonstrated the ability to develop resistance to most antibiotics recommended or used for treatment [5–7], suggesting the possibility of untreatable gonorrhea in the future [8–10]. The Centers for Disease Control and Prevention and the World Health Organization have labeled drug-resistant NG with threat levels of urgent and high, respectively, and identified a critical need for new antibiotic treatments [11–14]. Current guidelines recommend first-line dual antibiotic therapy that consists of intramuscular ceftriaxone combined with oral azithromycin [1, 15, 16]. Although this approach may delay the emergence of cephalosporin-resistant NG, the impending threat remains. Surveillance data demonstrate increasing minimum inhibitory concentrations (MICs) for NG to third-generation extended-spectrum cephalosporins and azithromycin in the United States, Canada, and Europe [13, 17–20]. Several therapeutic options are being evaluated for the treatment of gonorrhea, including dual combinations of established and novel antibiotics [5, 14, 21–23].

Gepotidacin (GSK2140944) is a novel triazaacenaphthylene bacterial type II topoisomerase inhibitor that is currently in development [24–29]. Gepotidacin selectively inhibits bacterial DNA replication by interacting in a unique way on the GyrA subunit of bacterial DNA gyrase and the ParC subunit of bacterial topoisomerase IV. Gepotidacin has in vitro activity against pathogens resistant to established antibacterials, including ciprofloxacin-resistant and -susceptible strains of NG [30, 31]. In this study, we evaluated the efficacy of single, oral doses of gepotidacin for treatment of uncomplicated urogenital gonorrhea.

## METHODS

### Study Design

**Figure 1.**
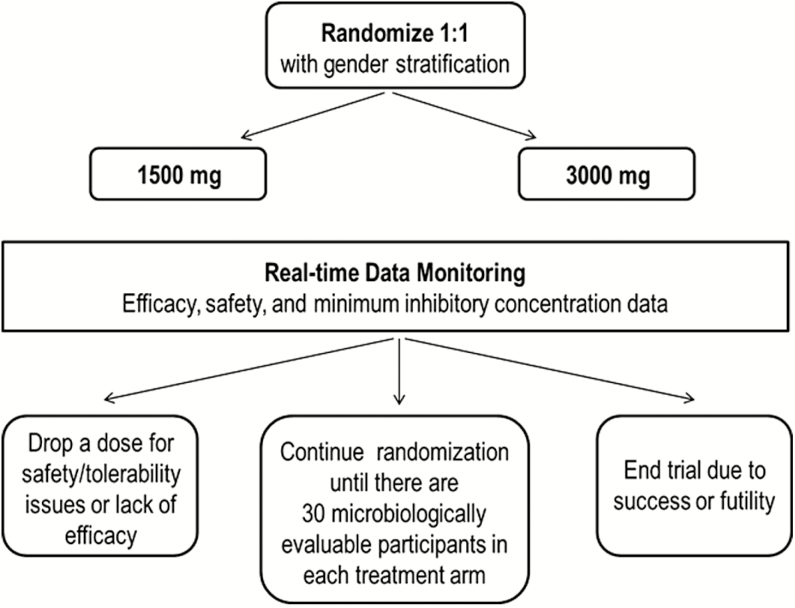
Study design.

This phase 2, randomized, multicenter, open-label, dose-ranging study was conducted at 12 sites (11 US sites and 1 UK site) from April 2015 to August 2016 to evaluate the efficacy, safety, tolerability, and plasma exposures of 2 single oral doses of gepotidacin in uncomplicated urogenital gonorrhea. Throughout the study, real-time sponsor-blinded reviews of safety data and NG isolate identification and susceptibilities by GlaxoSmithKline review teams were conducted. Additionally, an independent review team had the authority to recommend discontinuation of a dose, continue randomization as planned, or end the trial due to success or futility (Figure 1). The study was conducted in accordance with the International Council for Harmonisation of Technical Requirements for Pharmaceuticals for Human Use, Good Clinical Practice, and applicable country-specific requirements, including institutional review board and ethics committee approvals. Participants provided written, informed consent before any study procedures were performed.

### Participants

Men and nonpregnant, nonlactating women aged ≥18 years who had suspected uncomplicated urogenital gonorrhea, defined as the presence of purulent urethral or cervical discharge upon physical examination and a prior culture or nucleic acid amplification test (NAAT) positive for NG, a Gram stain positive for gram-negative diplococci from male urethral specimens, or reported sexual contact with a partner diagnosed with gonorrhea within the past 14 days were enrolled.

Exclusion criteria included a body mass index ≥40.0 kg/m^2^; hysterectomized women without a cervix; men diagnosed with epididymitis or orchitis; a medical condition or required medication that may have been aggravated by acetylcholinesterase inhibition; diagnosis of *Clostridium difficile* (CD) infection; liver disease; prespecified baseline electrocardiogram (ECG) abnormalities and cardiovascular conditions; disseminated gonococcal infections; antibiotic treatment within 14 days; or taking medications with a known risk of torsades de pointes. Participants agreed to abstain from or to use a male condom for any sexual activity from baseline through test-of-cure (TOC) to prevent reinfection.

### Participant Evaluation and Therapy

The total study duration was approximately 1 week with 2 study visits: baseline (day 1) and TOC (days 4–8). At baseline, a physical examination, assessment of vital signs, clinical laboratory tests, and ECGs were performed. Baseline microbiology procedures included collection of pretreatment urogenital swab specimens for NG culture from all participants; a Gram stain was performed on male urogenital specimens. From areas of known exposure, pretreatment pharyngeal and rectal swab specimens for NG culture were collected. Pretreatment urogenital swab or urine specimens for detection of NG and *Chlamydia trachomatis* (CT) by NAAT assay were obtained at baseline only. Eligible participants were stratified by gender and randomized 1:1 to receive a 1500-mg (3 × 500-mg capsules) or 3000-mg (6 × 500-mg capsules) single oral dose of gepotidacin administered open label with food at the clinic (Figure 1). Dose selection was based on in vitro and in vivo data and pharmacokinetic modeling, which indicated the low and high doses would provide systemic exposures to cover urogenital NG isolates with gepotidacin MICs of 0.5 µg/mL and 1 µg/mL, respectively. At 2 hours postdose, ECG and blood pharmacokinetic sample collection were performed. Based on blinded, real-time assessment of systemic exposures and ECG data from the first 37 participants, the sponsor safety review team determined postdose ECG and pharmacokinetic requirements could be safely removed.

Participants returned to the clinic at TOC for safety procedures similar to baseline and adverse event (AE) and concomitant medication data collection. At TOC, posttreatment urogenital swab specimens were collected for NG culture (and Gram stain for male urogenital specimens) from all participants who were positive for NG at baseline. Posttreatment pharyngeal and rectal swab specimens were also collected from anatomical sites that had a positive baseline NG culture. Standard-of-care treatment for CT was allowed after all TOC procedures were complete.

### Microbiological Evaluation

Gram stain, NG culture, and presumptive organism identification were performed at local laboratories. Recovered isolates of presumptive NG were sent to central laboratories (Q^2^ Solutions, Valencia, California, and University of Alabama, Birmingham) for confirmation of identification and susceptibility testing by agar dilution based on Clinical and Laboratory Standards Institute guidelines. Microbiological outcome (bacterial eradication, bacterial persistence, or unable to determine) and response (microbiological success or failure) for NG at TOC were determined based on predefined criteria. NG and CT NAATs were performed using the US Food and Drug Administration–cleared commercially available methods at local laboratories.

### Statistical Analyses

Participants were randomized into the study until approximately 30 microbiologically evaluable (ME) participants were included in each treatment arm (defined as all randomized participants who had NG isolated from baseline cultures of urogenital swab specimens, received either dose of gepotidacin, and returned for TOC). Assuming the true cure rate was 95%, a sample size of 30 ME participants was expected to provide at least 80% power to detect a difference of 15% (ie, cure rate of 95% under the alternative hypothesis and ≤80% under the null hypothesis) using a 1-sided binomial test at the 0.05 significance level.

The primary efficacy endpoint was the culture-confirmed bacterial eradication of urogenital NG at TOC (microbiological success). The null hypothesis (cure rate ≤80%) was tested using the binomial test for each dose level. To control the type I error rate of the final analysis at <0.05, a closed-testing procedure was used that required establishing significance at the higher dose before assessing the significance at the lower dose. The 2-sided 90% exact confidence interval (CI) was produced to reflect the 1-sided α = 0.05 [32]. If the 95% 1-sided CI for a dose level included 80%, then the null hypothesis was not rejected for that dose level. Subgroup analyses for age (18–64, 65–74, and ≥ 65 years) and gender were also performed. All analyses were conducted using SAS software, version 9.2 (SAS Institute, Inc., Cary, North Carolina).

Safety was assessed in all randomized participants who received either dose of gepotidacin. The safety secondary endpoint was evaluated by the analysis of AEs, including predefined cardiovascular and gastrointestinal AEs of special interest, vital sign assessments, laboratory values, physical examinations, and ECG parameters. The exploratory endpoints were bacterial eradication of pharyngeal and rectal NG when positive at baseline, microbiological characteristics of the isolates recovered, and a descriptive statistical summary of gepotidacin plasma concentrations. Gepotidacin plasma concentrations were also compared with phase 1 data from healthy volunteers.

## RESULTS

### Participant Population

**Figure 2.**
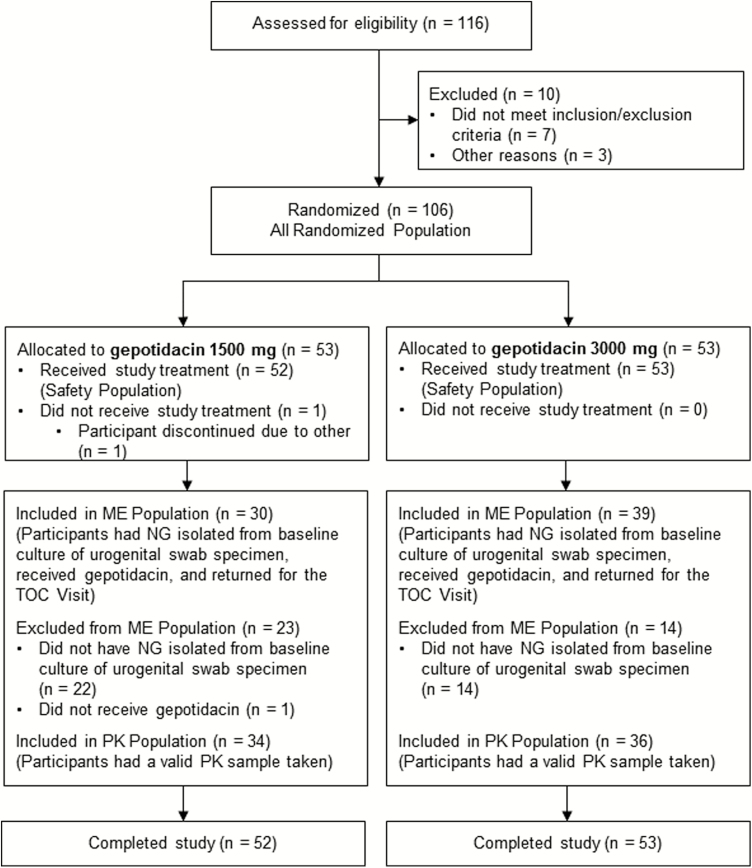
Participant disposition flow diagram. Abbreviations: ME, microbiologically evaluable; NG, *Neisseria gonorrhoeae*; PK, pharmacokinetic; TOC, test-of-cure.

A total of 106 participants were randomly assigned to 1500 mg (n = 53) or 3000 mg (n = 53) gepotidacin (Figure 2). One randomized participant (1500 mg) did not receive study treatment and 37 participants did not have NG isolated at baseline; thus, the ME population consisted of 69 participants (30 participants in the 1500-mg group and 39 in the 3000-mg treatment group).

The majority of participants were men (95%) and black (48%) with a mean age of 33.3 years; only 5 women were enrolled (Table 1). Based on NAAT results, 68 of 106 participants (64%) were positive for NG, 9 participants (8%) were positive for CT, and 4 (4%) were positive for both. The majority of Gram stain results for male urogenital specimens revealed the presence of white blood cells with gram-negative diplococci. In the ME population, NG isolates were identified in 69/69 (100%) urogenital, 2/69 (3%) pharyngeal, and 3/69 (4%) rectal specimens (Table 2). Of the urogenital NG isolates, 97% (67/69) were from men and 3% (2/69) were from women. Pharyngeal and rectal baseline NG isolates were recovered from men only.

**Table 1.** Demographics and Baseline Characteristics in the All Randomized Population

Characteristic	Gepotidacin 1500 mg (n = 53)	Gepotidacin 3000 mg (n = 53)	Total (N = 106)
Age, years			
Mean (range)	34.1 (18–63)	32.4 (18–69)	33.3 (18–69)
18–64	53 (100)	52 (98)	105 (>99)
65–74	0	1 (2)	1 (<1)
Sex			
Female	3 (6)	2 (4)	5 (5)
Male	50 (94)	51 (96)	101 (95)
Race			
Black	22 (46)	25 (50)	47 (48)
White	24 (50)	21 (42)	45 (46)
American Indian or Alaska native	1 (2)	1 (2)	2 (2)
Asian	1 (2)	1 (2)	2 (2)
Native Hawaiian or other Pacific Islander	0	1 (2)	1 (1)
White and black heritage	0	1 (2)	1 (1)
Nucleic acid amplification test^a^			
*Neisseria gonorrhoeae*			
Positive	31 (58)	37 (70)	68 (64)
Negative	22 (42)	14 (26)	36 (34)
Presence or absence cannot be determined	0	2 (4)	2 (2)
*Chlamydia trachomatis*			
Positive	6 (11)	3 (6)	9 (8)
Negative	47 (89)	48 (91)	95 (90)
Presence or absence cannot be determined	0	2 (4)	2 (2)

Data are presented as number (%) unless otherwise indicated. For the nucleic acid amplification test (NAAT) summary, the number of randomized participants in each respective treatment group or total was used as the denominator for the percentage calculation.

^a^The NAAT assay was reported at baseline for urogenital specimens only.

**Table 2.** Summary of *Neisseria gonorrhoeae* Recovery Rate at Baseline in the Microbiologically Evaluable Population

Specimen Source	Gepotidacin 1500 mg (n = 30)		Gepotidacin 3000 mg (n = 39)		Total (N = 69)	
	Number	Recovery Rate (%)	Number	Recovery Rate (%)	Number	Recovery Rate (%)
Urogenital						
Overall	30	100	39	100	69	100
Male	29	97	38	97	67	97
Female	1	3	1	3	2	3
Pharyngeal						
Overall	0	...	2	5	2	3
Male	0	...	2	5	2	3
Female	0	...	0	...	0	...
Rectal						
Overall	1	3	2	5	3	4
Male	1	3	2	5	3	4
Female	0	...	0	...	0	…

The numbers of participants in the treatment groups and total were used as denominators.

### Treatment Outcomes

The primary protocol-defined endpoint was met overall and in both treatment groups (Table 3).

**Table 3.** Summary of Bacterial Eradication by Culture for Urogenital, Pharyngeal, and Rectal *Neisseria gonorrhoeae* in the Microbiologically Evaluable Population

Microbiological Response–Microbiological Outcome	Gepotidacin 1500 mg	Gepotidacin 3000 mg	Total
Participants with urogenital NG at baseline	30	39	69
Microbiological success	29 (97)	37 (95)	66 (96)
1-sided 95% confidence interval	(85.1, 100)	(84.7, 100)	(89.1, 100)
1-sided *P* value	0.011	0.009	<0.001
Bacterial eradication	29 (97)	37 (95)	66 (96)
Microbiological failure	1 (3)	2 (5)	3 (4)
Bacterial persistence	1 (3)	2 (5)	3 (4)
Unable to determine	0	0	0
Participants with pharyngeal NG at baseline	0	2	2
Microbiological success	0	1 (50)	1 (50)
Bacterial eradication	0	1 (50)	1 (50)
Microbiological failure	0	1 (50)	1 (50)
Bacterial persistence	0	1 (50)	1 (50)
Participants with rectal NG at baseline	1	2	3
Microbiological success	1 (100)	2 (100)	3 (100)
Bacterial eradication	1 (100)	2 (100)	3 (100)

Data are presented as number (%) unless otherwise indicated. If either the count of successes or the count of failures was ≤5, then exact tests were conducted to compute *P* values.

Abbreviation: NG, *Neisseria gonorrhoeae*.

Microbiological success for urogenital gonorrhea was achieved by 97%, 95%, and 96% of participants (lower 1-sided exact 95% CI bound, 85.1%, 84.7%, and 89.1%, respectively) for the 1500-mg, 3000-mg, and combined dose groups, respectively. The ME population included 2 women, 1 in each treatment group, both of whom were microbiological successes for urogenital NG. Three participants (4%; 1 participant who received 1500 mg and 2 participants who received 3000 mg) were microbiological failures.

Analysis of subgroups was limited because the majority of study participants were in the age group of 18 to 64 years and were men. Only 2 participants had pharyngeal NG at baseline, both in the 3000-mg treatment group; 1 participant was a microbiological success and the other participant was a microbiological failure (Table 3). All 3 participants with rectal NG at baseline were microbiological successes, with 1 participant in the 1500-mg treatment group and 2 participants in the 3000-mg treatment group (Table 3). All microbiological failures received standard-of-care ceftriaxone 250 mg intramuscularly and azithromycin 1 g orally per national treatment guidelines.

### In Vitro Susceptibility Testing

In the ME population, of the 69 baseline urogenital NG isolates, 23/69 (33%), 19/69 (28%), and 14/69 (20%) were resistant to ciprofloxacin, penicillin, and tetracycline, respectively (Table 4). All of the baseline urogenital NG isolates were susceptible to cefixime, ceftriaxone, and spectinomycin.

**Table 4.** Urogenital Baseline *Neisseria gonorrhoeae* Antimicrobial Resistance for Selected Antimicrobials in the Microbiologically Evaluable Population

Antimicrobial Agent	n (%) of Resistant Isolates^a^		
	Gepotidacin 1500 mg	Gepotidacin 3000 mg	Total
Number of isolates	30	39	69
Cefixime	0	0	0
Ceftriaxone	0	0	0
Ciprofloxacin	8 (27)	15 (38)	23 (33)
Penicillin	10 (33)	9 (23)	19 (28)
Spectinomycin	0	0	0
Tetracycline	6 (20)	8 (21)	14 (20)

Isolates were defined as resistant based on Clinical and Laboratory Standards Institute (CLSI) guidelines. For cefixime and ceftriaxone, a susceptible only breakpoint was applied. Only drugs with CLSI breakpoints were included in the summary table. The counts in this table indicate the number of isolates, not the number of participants.

^a^Of the 2 pharyngeal *Neisseria gonorrhoeae* (NG) baseline isolates, susceptibility results were only available for 1 isolate, which was resistant to ciprofloxacin, penicillin, and tetracycline. Of the 3 rectal NG baseline isolates, 2 were resistant to tetracycline.

In the ME population, gepotidacin was active in vitro against the 69 urogenital NG isolates recovered, with overall MIC 50% and 90% values of 0.12 µg/mL and 0.5 µg/mL, respectively (Table 5), and MICs ranging from ≤0.06 to 1 µg/mL. Gepotidacin MICs ranged from ≤0.06 to 0.25 µg/mL against the pharyngeal (n = 2) and rectal (n = 3) NG isolates recovered.

**Table 5.** Summary of Baseline Minimum Inhibitory Concentration Results for Gepotidacin Against *Neisseria gonorrhoeae* in the Microbiologically Evaluable Population

Specimen Source Treatment	n	MIC Range		MIC_50_ (μg/mL)	MIC_90_ (μg/mL)
		Minimum (μg/mL)	Maximum (μg/mL)		
Urogenital					
Gepotidacin 1500 mg	30	≤0.06	1	0.12	0.5
Gepotidacin 3000 mg	39	≤0.06	1	0.25	0.5
Gepotidacin total	69	≤0.06	1	0.12	0.5
Pharyngeal					
Gepotidacin 1500 mg	...	...	...	...	...
Gepotidacin 3000 mg	2^a^	≤0.06	0.12	...	...
Gepotidacin total	2^a^	≤0.06	0.12	...	...
Rectal					
Gepotidacin 1500 mg	1	0.12	0.12	...	...
Gepotidacin 3000 mg	2	0.12	0.25	...	...
Gepotidacin total	3	0.12	0.25	...	…

The MIC_50_ and MIC_90_ values were not reported if n <10.

Abbreviations: MIC, minimum inhibitory concentration; MIC_50_, minimum inhibitory concentration required to inhibit the growth of 50% of organisms; MIC_90_, minimum inhibitory concentration required to inhibit the growth of 90% of organisms.

^a^Due to specimen contamination, susceptibility testing could not be reliably performed on the pharyngeal isolate from 1 participant in the 3000-mg treatment group at baseline.

All 3 participants who were urogenital microbiological failures had baseline NG isolates with a gepotidacin MIC of 1 µg/mL, which was the highest baseline gepotidacin MIC value observed in this study. Subsequent sequencing of the quinolone resistance-determining region (QRDR) of GyrA and ParC in these isolates revealed that all 3 isolates were quinolone resistant and had a preexisting D86 substitution due to a mutation in the *parC* gene, which is known to affect gepotidacin binding. Resistance also emerged between baseline and TOC for 2 of these urogenital microbiological failures, both in the high-dose 3000-mg treatment group. The gepotidacin MIC for both NG isolates from these 2 failures increased from 1 µg/mL to ≥32 µg/mL between baseline and TOC, and both TOC isolates were found to have an additional A92T substitution due to a mutation in the *gyrA* gene. For the 1 pharyngeal microbiological failure, the NG isolate had no change in gepotidacin MIC from baseline to TOC (0.12 µg/mL at both time points) and no mutations were observed in the QRDR of GyrA or ParC.

### Safety and Tolerability

There were no treatment-limiting AEs for either gepotidacin dose. Adverse events occurred in 27 of 52 (52%) and 34 of 53 (64%) participants in the 1500-mg and 3000-mg treatment groups, respectively (Table 6). No AEs led to study withdrawal and no deaths or serious AEs were reported. The most frequently reported AEs (>10% total) were diarrhea (27%), flatulence (23%), abdominal pain (15%), and nausea (13%). For nausea and diarrhea, the comparative relative risk 95% CIs were >1, suggesting a higher risk in the 3000-mg treatment group for these 2 AEs. The majority of AEs were mild to moderate in intensity, with 2 severe AEs, 1 each of flatulence (1500 mg) and dizziness (3000 mg).

**Table 6.** Adverse Event Overview and Summary of Common Adverse Events by Preferred Term in the Safety Population

AE Category	Gepotidacin 1500 mg (n = 52)	Gepotidacin 3000 mg (n = 53)	Total (N = 105)
Any AE	27 (52)	34 (64)	61 (58)
Related to study treatment	24 (46)	33 (62)	57 (54)
Leading to study withdrawal	0	0	0
Any serious AE	0	0	0
Common AEs by preferred term			
Diarrhea	9 (17)	19 (36)	28 (27)
Flatulence	14 (27)	10 (19)	24 (23)
Abdominal pain	6 (12)	10 (19)	16 (15)
Nausea	3 (6)	11 (21)	14 (13)
Fatigue	3 (6)	5 (9)	8 (8)
Dizziness	1 (2)	6 (11)	7 (7)
Hyperhidrosis	1 (2)	6 (11)	7 (7)
Abdominal discomfort	4 (8)	2 (4)	6 (6)
Feeling hot	1 (2)	4 (8)	5 (5)
Eructation	1 (2)	3 (6)	4 (4)
Feces soft	1 (2)	3 (6)	4 (4)
Somnolence	0	3 (6)	3 (3)

Data are presented as number (%). Common adverse events were defined as an AE with ≥5% incidence in any treatment group.

Abbreviations: AE, adverse event.

Cardiovascular AEs were reported in 2 participants (ECG ST segment elevation and palpitations) in the 1500-mg treatment group and 1 participant (tachycardia) in the 3000-mg treatment group. Investigators considered only the mild tachycardia as treatment related in a participant who had no underlying cardiovascular history. There were no dose-related trends observed for clinical laboratory evaluations, vital signs, or ECGs.

### Pharmacokinetics

**Figure 3.**
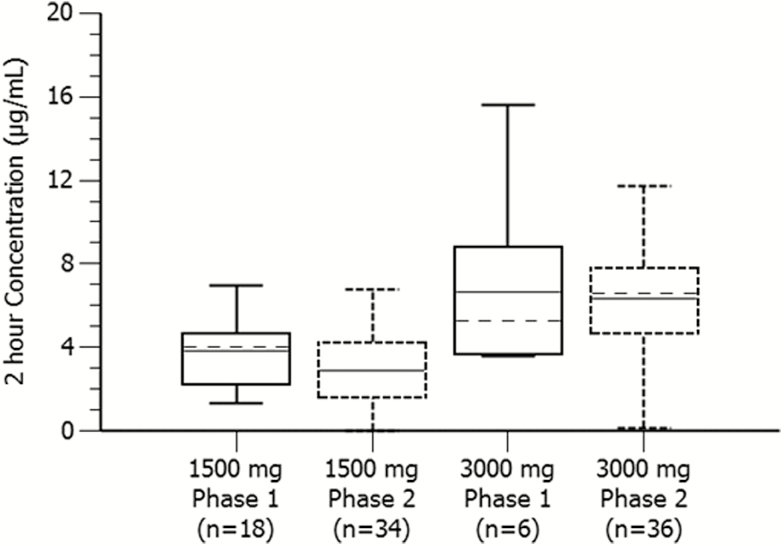
Comparison of gepotidacin single-dose plasma concentrations at 2 hours postdose between phase 2 participants with gonorrhea and phase 1 healthy volunteers. The solid midline is the mean and the broken midline is the median. The phase 1 data are from a single oral dose under fed conditions for 1500 mg and a single oral dose under fasted conditions for 3000 mg. The 1500-mg and 3000-mg phase 2 data are from the present study, which was a single oral dose administered with food.

The mean gepotidacin plasma concentrations (coefficient of variations) for the 2-hour postdose time point were 2.89 µg/mL (62.2%) and 6.35 µg/mL (42.9%) for the 1500-mg (n = 34) and 3000-mg (n = 36) single oral doses of gepotidacin, respectively, which was a dose proportional increase. A comparison of gepotidacin plasma concentrations between participants in the present study and phase 1 healthy volunteers showed comparable 2-hour postdose concentrations (Figure 3).

## DISCUSSION

As resistance to established antibiotics continues to rise for NG, new therapeutic options are needed to treat gonococcal infections. Gepotidacin is in development for the treatment of uncomplicated urogenital gonorrhea and is a first-in-class triazaacenaphthylene antibiotic with a novel mechanism of action. Gepotidacin given alone as a single oral dose (3 or 6 capsules) was ≥95% effective for bacterial eradication of culture-proven uncomplicated urogenital gonorrhea in this study. Gepotidacin may prove to be a therapeutic alternative for the treatment of uncomplicated gonorrhea. NG can demonstrate various resistance mechanisms [5–7]. To maintain the efficacy of gepotidacin and to help preserve the efficacy of other antibacterials, different dosing strategies for gepotidacin, including dual-agent therapy, may ultimately be needed.

For baseline urogenital NG isolates with gepotidacin MICs ≤0.5 µg/mL, the microbiological success rate was 100% in both treatment groups (n = 28, 1500 mg; n = 36, 3000 mg). Three urogenital microbiological failures were observed; all had isolates with the highest observed gepotidacin MIC of 1 µg/mL together with a common gene mutation known to affect gepotidacin binding; and all were men who have sex with men. Emergence of resistance to gepotidacin was observed for 2 of the urogenital microbiological failures. Further clinical development of gepotidacin for gonorrhea will need to consider dose optimization strategies and options to address NG isolates with higher gepotidacin MICs to minimize the potential for emergence of resistance.

With the single gepotidacin doses administered, there were no observed dose-limiting AEs. Blinded real-time reviews of safety data supported the randomization of participants to both the low- and high-dose groups throughout the study. The most frequently reported AEs were gastrointestinal in nature. Gepotidacin was administered with food in this study to minimize these effects. Such AEs have been observed previously for gepotidacin [26–29, 33] and are well known to be associated with some antibacterial therapies. CD infections were not reported in this study. Adverse events potentially associated with acetylcholinesterase inhibition, a known effect of gepotidacin based on in vitro competitive and reversible inhibition of the enzyme at clinically relevant concentrations [28], include dizziness, hyperhidrosis, and headache and were experienced by ≤6 participants in either treatment group. Potential dose-related trends were observed for some gastrointestinal and acetylcholinesterase inhibition AEs, but statistical significance could not be declared due to the small number of participants.

The effect of gepotidacin on cardiac conduction has been thoroughly evaluated [33], with results demonstrating a mild increased heart rate effect and a predicted mean QT prolongation of <15 milliseconds at plasma concentrations of 9 µg/mL, which is the highest plasma exposure expected clinically. With the high dose of 3000 mg in this study, mean 2-hour postdose plasma concentrations were approximately 6 µg/mL and were below the predicted QT threshold (<10 milliseconds), with no significant cardiac safety observations.

A comparison of gepotidacin plasma concentrations between participants in the present study and phase 1 healthy volunteers showed comparable 2-hour postdose concentrations (Figure 3). This phase 2 population consisted primarily of otherwise healthy participants; therefore, systemic exposures similar to those observed in phase 1 populations were expected.

One limitation of this study was the sample size, and a larger trial will be needed to confirm these results. Additionally, enrollment focused on participants with uncomplicated urogenital gonorrhea and few participants with pharyngeal or rectal gonorrhea were enrolled. Of these, 1 of 2 pharyngeal and all 3 rectal gonococcal infections were eradicated, indicating potential efficacy. Both men and women were enrolled; however, only 3% of the ME population were women. Future investigations will aim for a higher enrollment of women, but there will be limitations due to required reproductive health exclusions. Future studies will also collect sexual orientation for all participants to allow further assessment of resistance patterns. The ME population consisted of only US participants; thus, these study results may not reflect global NG epidemiology and resistance patterns. Last, coinfection with CT is common in individuals with gonorrhea [34]. Gepotidacin has not been shown to have activity against CT; thus, standard-of-care therapy was allowed after completing TOC assessments for participants who tested positive at baseline.

In conclusion, these results demonstrated that 1500-mg and 3000-mg single oral doses of gepotidacin were ≥95% effective for bacterial eradication of NG in adult participants with uncomplicated urogenital gonorrhea. Emergence of high-level resistance in 2 of 3 treatment failures is a concern. However, new therapies for drug-resistant NG are urgently needed. With additional clinical evaluation, gepotidacin may provide a much needed oral therapeutic option for uncomplicated urogenital gonorrhea as part of a new combination regimen.

## Notes

***Acknowledgments.*** We thank the participants, clinical staff, and investigators of this study for their participation, specifically Dr Susan Philip who enrolled the highest number of Microbiological Evaluable participants. We also thank the PPD team for their study contributions and Jodi Stahlman and Monica McGee at PPD for manuscript editorial and writing assistance. For laboratory contributions, we thank Q^2^ Solutions and the University of Alabama for their microbiology analyses and the GlaxoSmithKline genetics laboratory for their mutation analyses.

***Financial support.*** This work was supported by GlaxoSmithKline. This work was also supported in whole or in part with federal funds from the Office of the Assistant Secretary for Preparedness and Response, Biomedical Advanced Research and Development Authority, under an Other Transaction Authority Agreement (HHSO100201300011C).

***Potential conflicts of interest.*** The study was sponsored by GlaxoSmithKline (Collegeville, Pennsylvania). S. N. T. has received research support from Beckman Coulter, Becton Dickinson, Cepheid, GlaxoSmithKline (along with scientific advisory board and as a consultant), Hologic, Melinta, AstraZeneca, and Roche Molecular. B. E. B. has received research support from Melinta, AstraZeneca, and Entasis. C. A. T., N. E. S-O., C. R. P., A. R., M. H., and E. F. D. are employees at GlaxoSmithKline. Medical writing assistance was provided by PPD and funded by GlaxoSmithKline. Authors were not paid for their contributions. All other authors: No reported conflicts of interest. All authors have submitted the ICMJE Form for Disclosure of Potential Conflicts of Interest. Conflicts that the editors consider relevant to the content of the manuscript have been disclosed.

## References

[1] World Health Organization. WHO guidelines for the treatment of Neisseria gonorrhoeae. 2016 Available at: http://apps.who.int/iris/bitstream/10665/246114/1/9789241549691-eng.pdf?ua=1. Accessed 21 March 2017. 27512795

[2] Centers for Disease Control and Prevention. Sexually transmitted diseases surveillance 2016. Atlanta, GA: CDC, 2017 Available at: https://www.cdc.gov/std/stats16/CDC_2016_STDS_Report-for508WebSep21_2017_1644.pdf. Accessed 12 October 2017.

[3] European Centre for Disease Prevention and Control. Annual epidemiological report 2016–gonorrhea. Stockholm, Sweden: ECDC, 2016 Available at: https://ecdc.europa.eu/sites/portal/files/documents/Gonorrhoea%20AER_0.pdf. Accessed 25 July 2017.

[4] Hook EW, Handsfield HH Gonococcal infections in the adult. In: Holmes KK, Sparling PF, Stamm WE et aleds. Sexually transmitted diseases. 4th ed New York: McGraw-Hill, 2008:627–45.

[5] Unemo M, Shafer WM Antimicrobial resistance in *Neisseria gonorrhoeae* in the 21st century: past, evolution, and future. Clin Microbiol Rev 2014; 27:587–613. 24982323 10.1128/CMR.00010-14 PMC4135894

[6] Kirkcaldy RD, Ballard RC, Dowell D Gonococcal resistance: are cephalosporins next?Curr Infect Dis Rep 2011; 13:196–204. 21365384 10.1007/s11908-011-0169-9

[7] Unemo M, Golparian D, Nicholas R, Ohnishi M, Gallay A, Sednaoui P High-level cefixime- and ceftriaxone-resistant *Neisseria gonorrhoeae* in France: novel penA mosaic allele in a successful international clone causes treatment failure. Antimicrob Agents Chemother 2012; 56:1273–80. 22155830 10.1128/AAC.05760-11 PMC3294892

[8] Bolan GA, Sparling PF, Wasserheit JN The emerging threat of untreatable gonococcal infection. N Engl J Med 2012; 366:485–7. 22316442 10.1056/NEJMp1112456

[9] Ohnishi M, Golparian D, Shimuta K, et al Is *Neisseria gonorrhoeae* initiating a future era of untreatable gonorrhea?: Detailed characterization of the first strain with high-level resistance to ceftriaxone. Antimicrob Agents Chemother 2011; 55:3538–45. 21576437 10.1128/AAC.00325-11 PMC3122416

[10] Unemo M, Nicholas RA Emergence of multidrug-resistant, extensively drug-resistant and untreatable gonorrhea. Future Microbiol 2012; 7:1401–22. 23231489 10.2217/fmb.12.117 PMC3629839

[11] Centers for Disease Control and Prevention. Antibiotic resistance threats in the United States, 2013. Atlanta, GA: CDC, 2013 Available at: https://www.cdc.gov/drugresistance/threat-report-2013/pdf/ar-threats-2013–508.pdf. Accessed 31 May 2017.

[12] Centers for Disease Control and Prevention. Gonorrhea treatment guidelines: revised guidelines to preserve last effective treatment option. Atlanta, GA: CDC, 2013 Available at: https://www.cdc.gov/std/treatment/2010/gonorrhea-treatment-guidelines-factsheet.pdf. Accessed 20 March 2017.

[13] Kirkcaldy RD, Harvey A, Papp JR, et al *Neisseria gonorrhoeae* antimicrobial susceptibility surveillance—the Gonococcal Isolate Surveillance Project, 27 sites, United States, 2014. MMWR Surveill Summ 2016; 65:1–19. 10.15585/mmwr.ss6507a1 27414503

[14] Alirol E, Wi TE, Bala M, et al Multidrug-resistant gonorrhea: a research and development roadmap to discover new medicines. PLoS Med 2017; 14:e1002366. 28746372 10.1371/journal.pmed.1002366 PMC5528252

[15] Bignell C, Unemo M; European STI Guidelines Editorial Board 2012 European guideline on the diagnosis and treatment of gonorrhoea in adults. Int J STD AIDS 2013; 24:85–92. 24400344 10.1177/0956462412472837

[16] Workowski KA, Bolan GA; Centers for Disease Control and Prevention Sexually transmitted diseases treatment guidelines, 2015. MMWR Recomm Rep 2015; 64:1–137. PMC5885289 26042815

[17] Cole MJ, Spiteri G, Jacobsson S, et al Overall low extended-spectrum cephalosporin resistance but high azithromycin resistance in *Neisseria gonorrhoeae* in 24 European countries, 2015. BMC Infect Dis 2017; 17:617. 28893203 10.1186/s12879-017-2707-z PMC5594611

[18] Katz AR, Komeya AY, Kirkcaldy RD, et al Cluster of *Neisseria gonorrhoeae* isolates with high-level azithromycin resistance and decreased ceftriaxone susceptibility, Hawaii, 2016. Clin Infect Dis 2017; 65:918–23. 28549097 10.1093/cid/cix485 PMC6748320

[19] Soge OO, Harger D, Schafer S, et al Emergence of increased azithromycin resistance during unsuccessful treatment of *Neisseria gonorrhoeae* infection with azithromycin (Portland, OR, 2011). Sex Transm Dis 2012; 39:877–9. 23064537 10.1097/OLQ.0b013e3182685d2b PMC6746145

[20] Lefebvre B, Martin I, Demczuk W, et al Ceftriaxone-resistant *Neisseria gonorrhoeae*, Canada, 2017. Emerg Infect Dis 2018 Available at: https://wwwnc.cdc.gov/eid/article/24/2/17-1756_article. Accessed 29 November 2017. 10.3201/eid2402.171756 PMC5782888 29131780

[21] Hook EW 3rd, Golden M, Jamieson BD, et al A phase 2 trial of oral solithromycin 1200 mg or 1000 mg as single-dose oral therapy for uncomplicated gonorrhea. Clin Infect Dis 2015; 61:1043–8. 26089222 10.1093/cid/civ478

[22] Kirkcaldy RD, Weinstock HS, Moore PC, et al The efficacy and safety of gentamicin plus azithromycin and gemifloxacin plus azithromycin as treatment of uncomplicated gonorrhea. Clin Infect Dis 2014; 59:1083–91. 25031289 10.1093/cid/ciu521 PMC4271098

[23] Taylor SN, Marrazzo J, Batteiger B, et al A phase II trial of single-dose oral ETX0914 (AZD0914) for treatment of uncomplicated urogenital gonorrhea [abstract 5B5]. 2016 STD Prevention Conference (Atlanta). Sex Transm Dis 2016; 43:S147.

[24] Bax BD, Chan PF, Eggleston DS, et al Type IIA topoisomerase inhibition by a new class of antibacterial agents. Nature 2010; 466:935–40. 20686482 10.1038/nature09197

[25] Biedenbach DJ, Bouchillon SK, Hackel M, et al In vitro activity of gepotidacin, a novel triazaacenaphthylene bacterial topoisomerase inhibitor, against a broad spectrum of bacterial pathogens. Antimicrob Agents Chemother 2016; 60:1918–23. 26729499 10.1128/AAC.02820-15 PMC4776004

[26] Negash K, Andonian C, Felgate C, et al The metabolism and disposition of GSK2140944 in healthy human subjects. Xenobiotica 2016; 46:683–702. 26586303 10.3109/00498254.2015.1112933

[27] Tiffany CA, Hossain M, McDonald M, et al Safety and pharmacokinetics of single escalating oral doses of GSK2140944, a novel bacterial topoisomerase inhibitor [abstract F-1218]. 53rd Interscience Conference on Antimicrobial Agents and Chemotherapy (Denver) American Society for Microbiology, 2013 Available at: http://www.abstractsonline.com/Plan/ViewAbstract.aspx?sKey=5a00e178-5200-4888-bd14-54df807b1a86&cKey=e2bf6c2c-ce55-4645-97c2-085165aba0d2&mKey=7dd36e88-52c3-4ff1-a5df-1d00766558b8. Accessed 20 July 2017.

[28] Tiffany CA, Hossain M, McDonald M, Lerman S, Dumont EF Safety and pharmacokinetics of single escalating IV doses of GSK2140944, a novel bacterial topoisomerase inhibitor [abstract F-277]. 54th Interscience Conference on Antimicrobial Agents and Chemotherapy Washington, DC: American Society for Microbiology, 2014 Available at: http://www.abstractsonline.com/Plan/ViewAbstract.aspx?sKey=018d4f7b-adbb-4284-a1a7-bd3dacc88b1c&cKey=65c91e81-a211-4b2c-ba1d-fccfd39dd938&mKey=5d6b1802-e453-486b-bcbb-b11d1182d8bb. Accessed 20 July 2017.

[29] Tiffany CA, Hossain M, McDonald M, Lerman S, Dumont EF Safety and pharmacokinetics of repeat escalating IV doses of GSK2140944, a novel bacterial topoisomerase inhibitor [abstract F-278]. Interscience Conference on Antimicrobial Agents and Chemotherapy Washington, DC: American Society for Microbiology, 2014 Available at: http://www.abstractsonline.com/Plan/ViewAbstract.aspx?sKey=018d4f7b-adbb-4284-a1a7-bd3dacc88b1c&cKey=22532b0d-62d2-4c64-b47c-2856992644d4&mKey=5d6b1802-e453-486b-bcbb-b11d1182d8bb. Accessed 20 July 2017.

[30] Farrell DJ, Sader HS, Rhomberg PR, Scangarella-Oman NE, Flamm RK In vitro activity of gepotidacin (GSK2140944) against *Neisseria gonorrhoeae*. Antimicrob Agents Chemother 2017; 61:e02047–16. 28069643 10.1128/AAC.02047-16 PMC5328517

[31] Scangarella-Oman N, Hossain M, Dixon P, et al P2.38 Microbiological analysis from a phase ii study in adults evaluating single doses of gepotidacin (GSK2140944) in the treatment of uncomplicated urogenital gonorrhoea caused by *Neisseria gonorrhoeae*. Sex Transm Infect 2017; 93:A84–5. 10.1128/AAC.01221-18 PMC6256812 30249694

[32] Clopper C, Pearson S The use of confidence or fiducial limits illustrated in the case of the binomial. Biometrika 1934; 26:404–13.

[33] Hossain M, Zhou M, Tiffany C, Dumont E, Darpo B A phase I, randomized, double-blinded, placebo- and moxifloxacin-controlled, four-period crossover study to evaluate the effect of gepotidacin on cardiac conduction as assessed by 12-lead electrocardiogram in healthy volunteers. Antimicrob Agents Chemother 2017; 61:e02385–16. 28223381 10.1128/AAC.02385-16 PMC5404516

[34] Lyss SB, Kamb ML, Peterman TA, et al; Project RESPECT Study Group *Chlamydia trachomatis* among patients infected with and treated for *Neisseria gonorrhoeae* in sexually transmitted disease clinics in the United States. Ann Intern Med 2003; 139:178–85. 12899585 10.7326/0003-4819-139-3-200308050-00007

